# Thickness-Tunable PDMS-Based SERS Sensing Substrates

**DOI:** 10.3390/s25092690

**Published:** 2025-04-24

**Authors:** Diego P. Pacherrez Gallardo, Shu Kawamura, Ryo Shoji, Lina Yoshida, Binbin Weng

**Affiliations:** 1 School of Electrical and Computer Engineering, University of Oklahoma, Norman, OK 73019, USA; diego.p.pacherrez.gallardo-1@ou.edu; 2 Department of Chemical Science and Engineering, National Institute of Technology, Tokyo College, Tokyo 193-0997, Japan; s25803@tokyo.kosen-ac.jp (S.K.); shoji@tokyo-ct.ac.jp (R.S.)

**Keywords:** surface-enhanced Raman scattering (SERS), polydimethylsiloxane (PDMS), decamethylcyclopentasiloxane (D5)

## Abstract

Surface-enhanced Raman scattering (SERS) spectroscopy is an ultra-sensitive analytical method with the powerful signal-molecule detection capability. Coupling with the polydimethylsiloxane (PDMS) material, SERS can be enabled on a polymeric substrate for fast-developing bio-compatible sensing applications. However, due to PDMS’s high viscosity, conventional PDMS-SERS substrates are typically thick and stiff, limiting their freedom for engineering flexible micro/nano functioning devices. To address this issue, we propose to adopt a low viscosity decamethylcyclopentasiloxane (D5) solvent as a diluent solution. Via controlling the mixture ratio of D5 and PDMS and the spin-coating speed for deposition, this method resulted in a film of a well-defined thickness from sub-millimeter down to a 100 nm scale. Furthermore, thanks to the unsaturated Si-H chemical bonds in the PDMS curing agent, the PDMS film could effectively reduce the $Ag^{+}$ ions to Ag nanoparticles (NPs) directly bonding onto the substrate surface uniformly. Via adjusting the size and density of the AgNPs through reaction temperature and time, strong SERS was achieved and verified using R6G with the detection limit down to 0.1 ppm, attributed to the AgNPs’ plasmonic enhancement effect.

## 1. Introduction

Raman spectroscopy is a non-elastic optical-scattering-based analytical technique to non-destructively obtain highly specific “fingerprint” spectral information of target molecules, which has been widely used for the analysis of qualitative and quantitative molecular species and structural properties in various fields [1,2,3,4]. Because the intrinsic Raman scattering cross section is very weak, ranging from typical values of $10^{-30}$ ${\mathrm{cm}}^{2}$ to a higher end of $10^{-23}$ ${\mathrm{cm}}^{2}$, only a tiny fraction of incident photons undergo such a scattering pathway, meaning the conventional Raman method can only be used for analyzing samples with high concentrations. Instead, leveraging strong surface plasmonic field on metallic nanoparticles (NPs), such a surface-enhanced Raman scattering (SERS) could achieve a significant field enhancement [5], making this method have great potential for the ultrasensitive analysis of extremely low concentration analyte where trace-level detection is needed [6,7].

The effective SERS sensing process mainly depends on two mechanisms, including the electromagnetic enhancement (EM), and the chemical enhancement (CM). The aforementioned EM enhancement effect relies on the design and optimization of metallic NPs to enhance the localized surface plasmon resonance (LSPR). The resulting enhancement factor is of an order ranging from $10^{6}$ to $10^{14}$ [8,9] compared to conventional Raman spectroscopy. In contrast, the chemical mechanism (CM) enhancement effect stems from the physical interaction between the analyte and the LSPR field near the surface of metallic NPs. For effective surface-enhanced Raman scattering (SERS) detection, analyte molecules must be positioned in close proximity to the NP surface. This mechanism becomes particularly crucial when SERS analysis involves molecules with low physical affinity for the SERS substrate [10,11]. This is especially challenging with hydrophobic materials [12,13].

One strategy to address this issue is to use polydimethylsiloxane (PDMS), a widely recognized material known for its high hydrophobicity compared to typical hydrophilic SERS substrates [12,14,15]. This silicon-based polymer offers a promising approach for CM enhancement in such cases [16,17]. In addition to the affinity modification feature, the flexible PDMS substrates also offer high adaptability to be easily applied to irregular surfaces [18,19,20], which is unlike conventional rigid SERS substrates that often require complex and time-consuming manufacturing processes. In 2018, Zhao et al. [21] fabricated PDMS flexible substrates via silicon-assisted interfacial self-assembly transfer and Langmuir–Schaefer deposition technique for detection of pesticide residues. Similarly, in 2020, Li et al. [9] report a flexible SERS substrate consisting of Ag nanocubes onto PDMS film for detection of analyte on irregular surfaces as fish skin. In 2017, Kumar et al. [22] fabricated a flexible ultrasensitive SERS substrate based on silver (Ag)-coated on PDMS casting of elastomer PDMS on a Taro leaf as a template, showing high adhesion and hydrophobic behavior for detection of malachita green (MG). While PDMS can serve as a promising substrate material for advanced SERS technology, controlling its thickness still poses challenges, primarily due to its high viscosity, which limits its development for emerging fields such as wearable and bio-compatible applications. Additionally, it further complicates the integration of nanophotonic architectures into the substrate, which could otherwise provide substantial enhancements in light–matter interactions to further boost the SERS sensitivity.

In this article, therefore, we report our study on a new film fabrication method to produce PDMS-based SERS substrates with precisely controlled film thickness and the formation of embedded AgNPs. For lowering thickness, decamethylcyclopentasiloxane (D5) solvent was used as a diluent for adjusting the viscosity of PDMS solution. Together with tuning the spin-coater speed, we were able to define the film thickness from sub-millimeter down to a 100 nm scale. Further, regarding to activation of the SERS functionalization, the residual Si-H groups resulted from the PDMS curing process were leveraged as a reducing agent to donate electron to $Ag^{+}$ ions in the solution and reduce them to metallic AgNPs which nucleates and directly grows into Ag nanoparticles bonded on the cured PDMS elastomer surface. By optimizing the NPs morphology including size and distribution, we developed ultra-thin PDMS-SERS integrated substrates with enhanced sensing performance. Overall, this new straightforward fabrication approach allow us establish a robust material platform for designing future advanced SERS sensing technologies for potential applications in modern health, environmental monitoring, and defense sectors.

## 2. Materials and Methods

### 2.1. Chemical Agents and Experimental Supplies

For preparing the PDMS film, we use ${\mathrm{SYLGARD}}^{\mathrm{TM}}$ silicone elastomer base, and ${\mathrm{SYLGARD}}^{\mathrm{TM}}$ silicone elastomer curing agent manufactured from Trademark of The Dow Chemical Company (Midland, MI, USA). Sylgard 184 consists of a vinyl-terminated PDMS base with a viscosity of about 5100 cP and a curing agent containing a platinum catalyst and a crosslinking siloxane compound; using a 10 to 1 mix ratio, the viscosity (mixed) is about 3500 cP [23]. The base and curing agent are also marketed under Dow’s SYLGARD™ 184 Silicon Elastomer. The diluent solvent decamethylcyclopentasiloxane 97% (D5) was purchased from Sigma-Aldrich (St. Louis, MO, USA). For AgNPs synthesis and Raman analysis, we ordered the high purity AgF (>99.9%) and Rhodamine 6G (95%) power, and the pure Ethyl alcohol (>99.5%) from Sigma Aldrich as a diluent. Lastly, the uncoated Soda Lime Glass substrates with the size of 25(W) × 25(H) × 1.1(T) ${\mathrm{mm}}^{3}$ and transmittance > 90% were purchased from MSE Supplies (Tucson, AZ, USA), and used as holding wafers to fabricate PDMS-SERS substrates.

### 2.2. Preparation of Thickness Controllable PDMS Films

**Figure 1:**
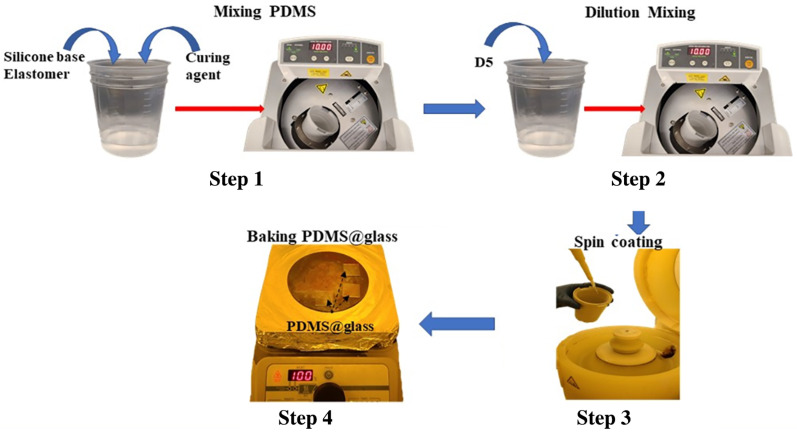
Process flow for preparing D5-diluted PDMS films including: Step 1 to mix the PDMS components in a 10:1 ratio of silicone elastomer to curing agent; Step 2 to dilute the solution with D5 solvent; Step 3 to spin-coat the D5-diluted PDMS onto a glass slide to control the final film thickness; Step 4 to bake the deposited PDMS on glass to cure the film.

To fabricate the PDMS base film, two main methods exist: ultraviolet (UV) light curing and thermal curing. In this study, we selected the latter for its quite obvious advantages, including the wider availability of materials in the market and its simpler, more straightforward process compared to the UV curing process, which requires a specialized UV light source and often the use of a glovebox. The thermal curing process of PDMS proceeds via a hydrosilylation reaction, in which Si-H groups from the cross-linker curing agent react with vinyl groups of the base polymer, resulting a cross-linked transparent PDMS elastomer product. The systematic study of this chemical reaction process has been reported in detail in previous works [24,25]. As shown in Figure 1, the PDMS polymeric solution was prepared by combining the elastomer base and curing agent in a 10:1 ratio, respectively, followed by mixing and degassing using a Thinky USA AR-100 conditioning mixer (Laguna Hills, CA, USA) at 2000 rpm for 10 min Figure 1 (step 1). After the initial mixing step, the D5 solvent was added as a diluent to the solution in PDMS:D5 weight ratios of 1:1, 1:2, 1:4, 1:8, and 1:16, prior to spin-coating onto a glass wafer.

There are two major reasons for choosing D5 as a diluent. Firstly, D5 has a very low viscosity (around 4.0 cSt at 25 °C) [26], significantly lower than that of uncured PDMS (around 3500 cP or 3400 cSt for the 10:1 mixed elastomer without the D5 dilution) [23]. When added to a PDMS solution, D5 reduces the overall viscosity by diluting the concentration of the high-viscosity polymer chains, effectively thinning the mixture. Second, D5 is a cyclic siloxane sharing a similar silicone-oxygen (Si-O) backbone structure with PDMS. Such structural similarity ensures that D5 is chemically compatible with PDMS, allowing it to mix homogeneously without causing phase separation or disrupting the polymer’s integrity. In addition, D5 also carries other advantageous features including the non-reactive nature as well as the low boiling point and volatility which ensure a minimal residue in the final thermally cured PDMS film. As a matter of fact, the effectiveness of using D5 to dilute PDMS has been studied previously [27].

Following the D5 mixing process, each resulting solution was further blended in the AR-100 mixer using the same parameters as the initial step. The spin-coater was then utilized to deposit the D5-diluted PDMS solutions onto glass wafers at various spin speeds. To investigate the optimal thickness dependence on coating speed, we initially fixed the PDMS:D5 mixing ratio at 1:3 and varied the spin speeds from 1000 rpm to 5000 rpm for 1 min. Subsequently, we fixed the spin speed at 3000 rpm and adjusted the mixture ratios from 1:1 to 1:5 to examine the optimal ratio dependence. A minimum volume of 200 μL of each mixture with different ratios was prepared for the coating experiments on the glass wafers. Following spin-coating, each film was soft-baked at 100 °C for 20 min to fully cure the coated films.

### 2.3. AgNPs Reduction to Enable the SERS Effect on PDMS Substrates

The mechanism by which PDMS facilitates the reduction of Ag ions to form AgNPs typically involves the reducing capability of residual reactive groups within the PDMS matrix, particularly the Si-H (hydride ion) groups present in the curing agent used during PDMS synthesis. During the curing process, the hydrosilylation reaction happens between the vinyl groups and Si-H groups, cross-linking the polymer into a solid elastomer. However, not all Si-H groups are consumed in this reaction. These residual Si-H groups are then exposed to a $Ag^{+}$ ion source via immersing the PDMS film into $Ag^{+}$ salt (e.g., silver nitrate $AgNO_{3}$ or silver flouride AgF solutions). The hydride ($H^{-}$) from Si-H can donate electrons to $Ag^{+}$ ions, reducing them to metallic $Ag^{0}$, which then nucleates into AgNPs and directly grow on the PDMS cross-linked film matrix. The general reaction can be expressed as:

$$2Ag+R_{3}S\mathrm{i-H}\to 2Ag^{0}+R_{3}Si^{+}+H^{+}$$

Here, $R_{3}S\mathrm{i-H}$ represents the Si-H group within the PDMS matrix, where R denotes the organic substituents (e.g., methyl groups in PDMS). In our work, we have studied both $AgNO_{3}$ and AgF-based $Ag^{+}$ ion solutions, and decided to use AgF due to the fact that $F^{-1}$ ions serve as an very effective catalyst that can significantly speed up the $Ag^{+}$ reduction process with the PDMS film. Similar study has been also reported in other works [28,29].

**Figure 2:**
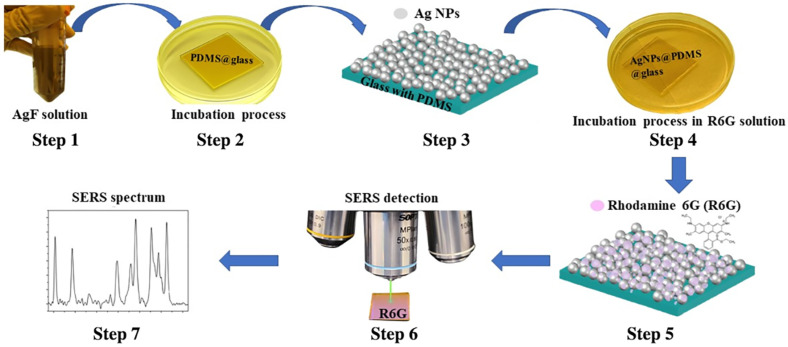
Preparation and testing of the PDMS-SERS substrate including Step 1 is to prepare the ethanolic AgF solution; Step 2 is to incubate the PDMS film in the $Ag^{+}$ solution; Step 3 is to clean and dry the AgNP deposited PDMS film after the incubation; Step 4 is to incubate the AgNPs deposited PDMS substrate in the diluted R6G analyte solution; Step 5 is to wash and dry the R6G molecule coupled PDMS-SERS substrate; Step 6 is to conduct the confocal Raman spectrum measurement; and Step 7 is to process and analyze the data from the measured Raman results.

To take advantage of the residual Si-H groups, the fresh PDMS film were incubated right after the curing process in the ethanolic AgF solution at room temperature. To prepare the AgF solution, we dissolved 31.72 mg of AgF powder in 25 mL of ethanol, then ultrasonic for 3 min until the powder is dissolved to obtain a solution of 10 mM concentration. We immersed every sample in 5 mL of solution for different reaction times, namely, from 1 min to 20 min and incubated at room temperature. During this process, a color change transiting from originally transparent film to yellowish can be clearly observed due to the nucleation of AgNPs. After the completion of reaction process, each sample was taken out and rinsed with ethanol (the same solvent used to prepare the $Ag^{+}$ salt solution), and then dried with nitrogen flow, as shown in Figure 2. Subsequently, multiple techniques were employed to characterize the material properties, including a J.A. Woollam VASE32 spectroscopic ellipsometer (Lincoln, NE, USA), a JEOL 2010-F Field Emission high-resolution transmission electron microscopy (TEM) (Akishima, Tokyo, Japan) and a Shimadzu UV-1601 UV-vis spectrometer (Kyoto, Japan).

In order to verify the effective SERS function of the as-prepared AgNP embedded PDMS substrates, the Raman sensing experiments were then conducted following the process as illustrated in Figure 2. The AgNP embedded PDMS substrates were incubated in the Rhodamine 6G (R6G) solution with different diluted low concentrations, e.g., 1 parts-per-million (ppm), with a time duration of 20 min to effectively bind the R6G uniformly cross the surface of the substrate. Having the dried substrates, Raman spectra were collected using a RTS-Mini confocal Raman spectrometer made by Zolix by focusing the excitation laser of the wavelength at 532 nm through a 50× long working distance objective lens. The original power from the laser source is set to be 15 mW, but to avoid the burning damage on the SERS substrates, an optical filter with 90% reduction has been applied for all the measurements. At the same time, to ensure the good signal quality, our standard Raman measurement setup choose to use the high spectral resolution grating of 1800 g/mm (line per millimeter), five accumulations and 1 s acquisition time. The collected Raman spectra were then processed via a polynomial background fitting method to remove the background signal and thus isolate the true Raman peaks for enhancing the analysis quality. The analysis of the SERS film’s performance will be elaborated in the later section.

## 3. Results and Discussion

### 3.1. The Study of Thickness Control via Mixing Ratios and Spin-Coating Speeds

In this work, the PDMS films were deposited onto glass wafers using the spin-coating method, which is a widely adopted and effective technique for creating thin and uniform films from a liquid solution on a substrate. This process is governed by two critical parameters that determine the final film thickness: centrifugal shear forces and viscous flow. The centrifugal shear forces can be adjusted by varying the spin-coating speed, while the viscous flow is constrained by the physical viscosity of the solution.

**Figure 3:**
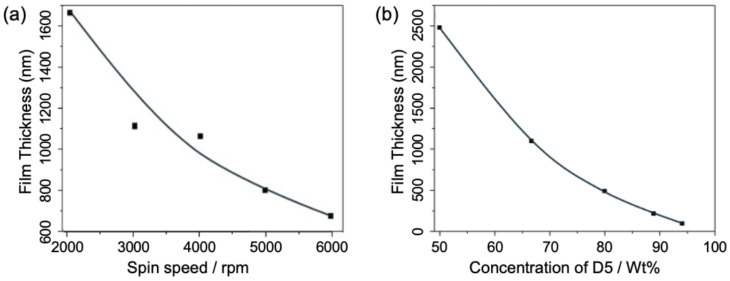
(**a**) The thickness as the function of spin-coating speed for film deposition with the D5:PDMS weight ratio fixed at 2:1; and (**b**) PDMS film thickness dependency with varying D5:PDMS weight ratios (wt%) at a fixed speed of 3000 rpm.

Consequently, we first examined the influence of shear forces on film thickness by controlling the spin-coating speed during the deposition process. With the D5:PDMS weight ratio fixed at 2:1—a moderate value within the tuning range—we varied the spin-coater speed from 2000 rpm to 6000 rpm in increments of 1000 rpm. As shown in Figure 3a, the film thickness decreased significantly from 1637 nm to 671 nm as the spin speed increased from 2000 rpm to 6000 rpm. As noted earlier, this reduction occurs because the PDMS:D5 mixture experiences greater shear forces at higher spin speeds, enhancing the outward flow of the solution from the substrate and resulting in a thinner film. Additionally, as spin speed increased, the liquid PDMS film thinned rapidly at lower speeds, but the rate of reduction slowed in the higher speed range, starting from 4000 rpm. This is attributed to surface tension beginning to counteract the centrifugal shear forces. In other words, as the film becomes very thin, the viscosity of the remaining solution on the wafer resists further flow, limiting additional thickness reduction. Thus, to achieve even thinner films at this stage, reducing the solution viscosity by adjusting the D5:PDMS mixing ratio becomes critical.

To evaluate the effect of the D5:PDMS mixing ratio, D5 solvent was blended with PDMS at weight ratios of 1:1, 1:2, 1:4, 1:8, and 1:16. All samples underwent spin-coating at a fixed speed of 3000 rpm, followed by curing at 100 °C for 20 min. Thickness measurements, depicted in Figure 3b, demonstrate the relationship between film thickness and the PDMS:D5 mixing ratio. As observed, an increase in the D5 weight ratio within the PDMS solution led to a corresponding reduction in film thickness, ranging from approximately 2500 nm at a 50% D5:PDMS ratio to about 95 nm at a 94% D5:PDMS ratio. The resulting curve displays a parabolic trend, characterized by a near-linear, substantial decrease between 50% and 70% D5:PDMS ratios, followed by a more gradual decline as the D5 proportion increased further. This pattern is anticipated, as the change in viscosity becomes less pronounced when the low-viscosity D5 dominates the mixture. Nevertheless, the D5 solvent proves highly effective in lowering the viscosity of the PDMS solution, facilitating a broad thickness tunability.

Overall, the results presented in this section show the critical roles of both the spin-coating speed and the D5:PDMS mixing ratio parameters in defining the final PDMS film thickness. Via optimizing both parameters, we can successfully achieve a broad thickness control range from micrometers to sub-hundred nanometers.

### 3.2. The Study of AgNPs Reduction Control via the Reaction Time and Temperature Parameters

As enabling chemical sensing functionality on the PDMS film is another major objective of this work, in this part, we will present the study of the control of the simple $Ag^{+}$ ion reduction method to achieve the direct growth of SERS-active AgNPs on the PDMS film. As mentioned previously, our method is to take the advantage of the residual Si-H groups on the freshly prepared PDMS substrate, which can be oxidized to silanol (Si-OH) when exposed to the AgF solution containing $Ag^{+}$ ion source. $Ag^{+}$, as a result, are reduced to metallic $Ag^{0}$, which nucleates and grows into AgNPs. It is also worth noting that the process occurs in situ, meaning the AgNPs formation happens directly on the PDMS matrix without requiring additional reducing agents. In addition, the hydrophobic nature of PDMS can also influence the nucleation and growth of AgNPs, often confining them to the surface or near-surface regions.

While several experimental factors can affect the $Ag^{+}$ reduction process, such as the reaction temperature and time, the concentration of Si-H groups and $Ag^{+}$ ions, as well as the base solution choices (e.g., water or organic solvents), in our study, we found the reaction time serves as the key parameter if Ag ion solution is made by AgF and ethanol, which allows us to have a controllable dynamic range to tune the AgNPs deposition results. Due to the hydrophobic nature of PDMS, it has a low surface energy that repels polar solvents like water. Thus, ethanol is our solvent choice which not only has good solubility with the AgF salt, but is also less polar than water having much better wetting properties on PDMS for promoting the $Ag^{+}$ ion diffusion to the PDMS surface.

**Figure 4:**
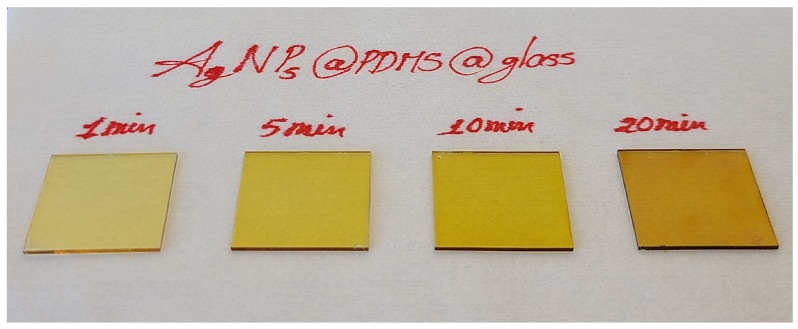
Four PDMS samples embedded with the directly deposited AgNPs chemically synthesized in a 10 mM AgF solution for reaction times of 1, 5, 10, and 20 min.

Regarding the salt choice, although the $AgNO_{3}$ salt is relatively stable and generally widely-used, its reaction with Si-H unfortunately is very slow, taking hours to form sizable AgNPs, and thus requires raising the reaction temperature to shorten the time span down to the sub-hour level. It not only further complicates the process and is still time consuming in contrast to the use of AgF whose $Ag^{+}$ reduction process with PDMS is much more fast and can achieve AgNPs of a very high density in a few minutes simply at room temperature. This is clearly illustrated in Figure 4, which displays four AgNP-deposited PDMS films on 1 × 1 inch^2^ glass wafers. As the reaction time increased from 1 min to 20 min, the freshly prepared transparent PDMS films transitioned to yellow, with the color darkening as the reaction time extended. This indicates successful control of $Ag^{+}$ reduction and AgNP deposition on the PDMS surface. The variation in color qualitatively reflects an increase in the density of deposited NPs. Moreover, all four samples exhibit highly uniform color across the wafer, suggesting a consistent distribution of AgNPs—an essential factor for the future sensor development.

**Figure 5:**
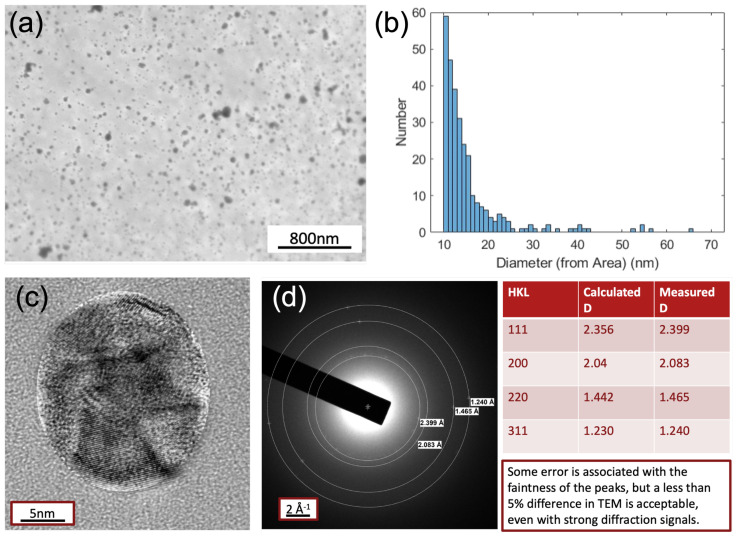
(**a**) Top-view TEM image of AgNP-deposited PDMS surface at the scale of 800 nm; (**b**) the statistic analysis of the size distribution; (**c**) the contrast enhanced imaging of the single AgNP; (**d**) the electron diffraction patterns for revealing the crystalline information of the AgNP.

**Figure 6:**
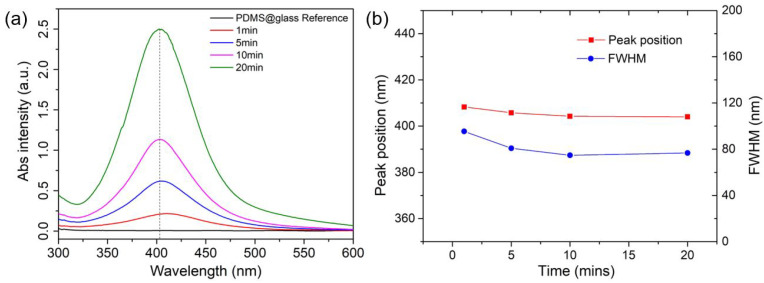
(**a**) Absorbance spectra of AgNPs-embedded PDMS substrates; (**b**) the comparison of the peak positions and full width of half maximum (FWHM) values of the collected spectra.

In order to obtain the direct and clear insights of the AgNPs deposited on the PDMS films, TEM measurements have been conducted. The TEM’s top-view figure is presented in Figure 5a. The statistical analysis of the size distribution in Figure 5b shows that the majority of the size distribution of AgNPs falls at around 10 nm with a distribution tail decreasing quickly for the larger sizes. This matches well with the resonant peak position observed from the UV–vis absorption spectra to be discussed in the following Figure 6 section. Furthermore, the contrast enhanced crystallinity measurement and electron diffraction patterns illustrated in Figure 5c,d, also indicates that the polycrystalline nature of AgNPs with a cluster of orientations in (111), (200), (220) and (311).

It is known that when AgNPs possess appropriate sizes, they can exhibit strong localized surface plasmon resonance (LSPR), a phenomenon in which the free electrons on the NP surface oscillate in response to incident light. This effect can significantly enhance the Raman sensing capability (i.e., SERS) when aligned with the proper excitation wavelength. The UV–vis spectrometer was then utilized to characterize the plasmonic properties of the AgNP-deposited PDMS film, inferring their NP morphology information accordingly. Figure 6 presents the UV–vis absorption spectra of a pure PDMS sample and four AgNP-deposited PDMS samples, each subjected to different $Ag^{+}$ reduction times (1, 5, 10, and 20 min) in a 10 mM AgF solution.

The pure PDMS sample (PDMS@glass) serving as the reference exhibits negligible absorbance across the 300–800 nm range, confirming its optical transparency. In contrast, the AgNP-deposited samples display distinct LSPR peaks centered around 400 nm. As the reduction time increases from 1 to 20 min, the LSPR peak intensity rises significantly, with absorbance increasing from approximately 0.5 to 2.5, reflecting a higher density of AgNPs on the PDMS surface, consistent with the observed darkening of the film color from light to deep yellow. Additionally, as depicted in Figure 6b, no red shift and peak broadening (i.e., FWHM) trends are observed from the samples with longer reaction times, suggesting a self-constrained $Ag^{+}$ ion reduction process on the PDMS surface. This is likely attributable to the limited availability of Si-H groups at each reaction site, which restricts the NP size increase and also reduces the likelihood of the further NP aggregation. This unique property provides a significant advantage to the method, as many other solution-based NP synthesis techniques commonly face these challenges, making precise control difficult to achieve. Overall, these results demonstrate the successful tuning of AgNP density and size through reaction time, as to optimize the plasmonic properties of AgNP-deposited PDMS for the possible enhanced SERS sensing applications.

### 3.3. The Raman Sensing Effect Verification of the SERS-PDMS Substrates

**Figure 7:**
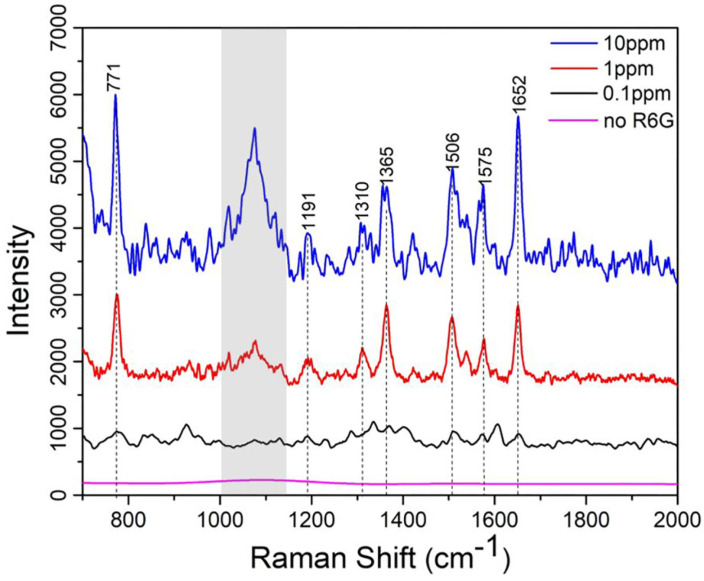
The concentration-dependent Raman spectra of R6G molecules incubated on the SERS active PDMS substrates: 0 ppm, 0.1 ppm, 1 ppm and 10 ppm.

In this final section, to evaluate the SERS performance of the AgNP-embedded PDMS substrates, Raman measurements were conducted using R6G as the probe dye molecule at three different concentrations: 10 ppm, 1 ppm, and 0.1 ppm. The AgNP-embedded PDMS samples were incubated in the respective R6G solutions for 20 min to ensure sufficient adsorption of the dye onto the substrate surface, followed by thorough ethanolic rinsing to remove any unbound molecules. The SERS spectra, as shown in the Figure 7, reveal distinct Raman peaks corresponding to R6G’s characteristic vibrational modes at approximately 771, 1191, 1310, 1361, 1506, 1575, and 1652 ${\mathrm{cm}}^{-1}$, which are associated with the C-C stretching, C-H bending, and aromatic ring deformation of the molecule. The signals within the grey color blocked band represent background noise originating from the microscope system and should be disregarded during analysis. As shown, at 10 ppm (blue curve), the peaks exhibit the highest intensity, reflecting a strong SERS enhancement due to the high concentration of R6G molecules interacting with the AgNPs’ LSPR modes. As the concentration decreases to 1 ppm (red curve) and 0.1 ppm (black curve), the peak intensities reduced progressively, yet the signature peaks of R6G still remain detectable even at 0.1 ppm, showing the limit of detection of the substrate prepared via the method presented in this work.

Furthermore, it is noted that the SERS signal intensity does not present a very good linear dependence on the analyte concentration. This can be attributed to several factors commonly observed in SERS experiments: (1) At higher analyte concentrations, the analyte molecules may aggregate, which can change their orientation or distance from the AgNP surface, reducing the enhancement effect and altering signal intensity; (2) The SERS enhancement is highly dependent on “hot spots”—localized regions of strong electromagnetic field enhancement. As analyte concentration increases, not all molecules may be adsorbed in these regions. This can also lead to a non-linear response.

In addition, we want to point out that the SERS spectrum for the 10 ppm sample appears noisier than that of the 1 ppm sample, despite both being measured using identical Raman settings and substrate preparations. While this may seem counterintuitive, several factors could contribute to this result. One possibility is that at higher concentrations (e.g., 10 ppm), the R6G molecules may begin to aggregate or form multilayers on the AgNP surface. This can lead to reduced enhancement efficiency due to a loss of optimal analyte–nanoparticle proximity (i.e., molecules not residing within the “hot spots”) and result in broader or less well-defined spectral features, thereby increasing apparent noise. Another possibility is that signal saturation or fluorescence background from densely packed R6G molecules might obscure or distort the Raman signal, particularly at higher concentrations, making the spectra appear noisier. Slight sample inhomogeneities or local field screening at higher coverage may also contribute to reduced reproducibility and elevated noise.

**Figure 8:**
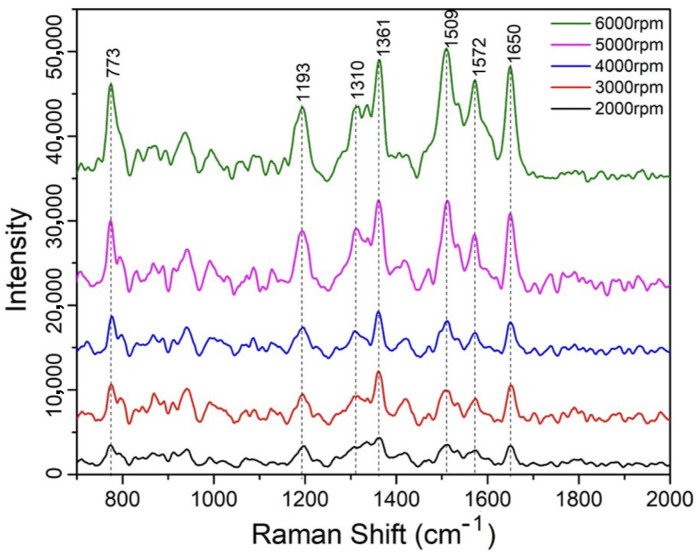
Thickness-dependent SERS spectra of 1 ppm R6G concentration on samples coated with PDMS substrates under different spin-coating speeds.

We then investigated the thickness-dependent SERS signal of the D5 diluted PDMS-based SERS substrates using the 1 ppm R6G analyte. Specifically, we prepared a D5:PDMS mixed solution with a ratio of 1:2, and spun coated five samples under different spin speeds, from 2000 rpm to 6000 rpm with a 1000 rmp incremental step. After the thermal curing process at ${100\;}^{\circ }$C for 20 min, these samples were then incubated in R6G solution at a concentration of 1 ppm for another 20 min and dried for conducting Raman signal measurement. As shown in Figure 8, the Raman spectra (800 to 2000 ${\mathrm{cm}}^{-1}$) reveal a clear trend: SERS intensity increases as spin speed rises, corresponding to thinner PDMS films. For instance, the 1361 ${\mathrm{cm}}^{-1}$ peak (aromatic C-C stretching) intensity increases by around 4-fold from 2000 rpm (thickest) to 6000 rpm (thinnest). In other words, the SERS sensing effect increases with the reduction of the PDMS thin film thickness.

We attribute this enhancement to greater AgNP exposure in thinner films, which increases the density of SERS-active hot spots accessible to R6G molecules. With lower spinning speed, more void space that the D5 created after its evaporation through the thermal curing process will remain in the body of the film which makes it harder for the R6G molecule to diffuse and couple with the reduced AgNPs formed around those spaces that are within the PDMS body matrix. This is especially true due to the high hydrophobicity nature of the PDMS film. However, when it comes to the thinner PDMS film, most void space created by the evaporated D5 solution stays on the top surface of the film, therefore, it will effectively expose more AgNPs to R6G molecules, leading to the enhanced Raman sensing signal as a result.

**Figure 9:**
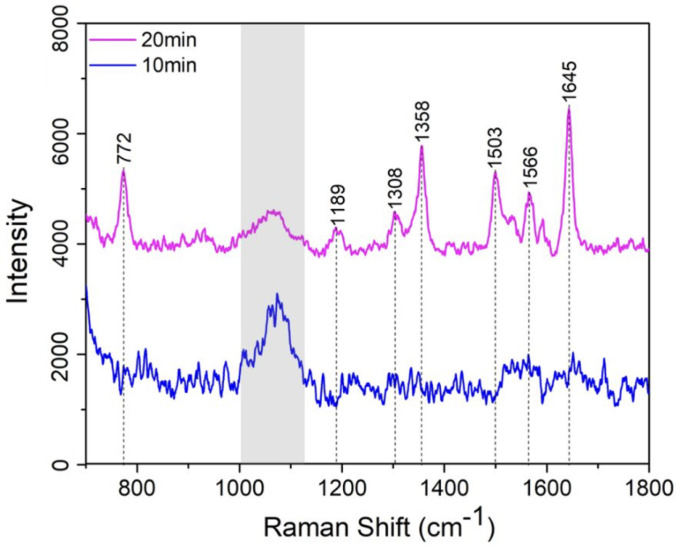
SERS spectra of 1 ppm R6G concentration under incubation time at 10 and 20 min (note: the peak in the grey shadow only comes from the silicon substrate).

Lastly, we also found that a prolonged incubation time for R6G is essential for this specific analytical study. As shown in Figure 9, to evaluate the time-dependent coupling efficiency of R6G with the SERS-active PDMS substrate, the Raman spectra of 1 ppm R6G on AgNP-embedded PDMS substrates are compared for incubation times of 10 min (blue curve) and 20 min (pink curve). While the 20 min incubation results in noticeably higher peak intensities, the 10 min one shows no signature peaks, indicating insufficient R6G adsorption.

We suggest this phenomenon is due to a competing dynamic: the hydrophobic nature of PDMS restricts R6G interaction with the substrate surface, whereas the synthesized AgNPs exhibit strong electrostatic affinity, promoting effective R6G bonding. A sufficient incubation period is thus critical to balance these competing mechanisms and ensure optimal R6G adsorption on the AgNPs-embedded PDMS substrate for SERS detection. This can be understood by the adsorption kinetic process governed by a combination of diffusion-limited transport and surface interaction dynamics. We uses the kinetic model based on Langmuir-type adsorption [30], which is expressed as: $d\theta /dt=k_{a}C\left(1-\theta \right)-k_{d}\theta$ in which, θ is described as the surface coverage of R6G molecules on the AgNPs, C is the solution concentration of R6G, and ka and kd are the adsorption and desorption rate constants, respectively. Due to the ultra-low concentration of R6G and hydrophobic surface nature of PDMS film, the adsorption process is initially far from saturation and the desorption is also negligible over the initial incubation times. Thus, the equation can be simplified as $\theta =k_{a}Ct$. Obviously, the SERS signal is proportional to the θ which also means that the signal increases approximately linearly with time in the early regime. However, in our case, due to the hydrophobic PDMS surface, initial adsorption is inefficient due to the uneven distribution of R6G in the solution (lower concentration C near the PDMS surface) and the non-linear ka coefficient at the initial phase, and thus creating a lag of the absorption process. After passing a critical time threshold, which is at least 10 min in our case, local concentration near AgNPs may begin to increase due to weak accumulation effects or improved surface affinity at localized AgNPs sites. Thus, the system could transition into a more rapid adsorption phase, resulting in a nonlinear amplification of the surface coverage and consequently the enhanced SERS signal as shown in Figure 9.

Although this setup may not be time-efficient for the SERS effect study due to PDMS’s hydrophobicity, it could imply a unique advantage in mitigating surface fouling—a common challenge with traditional hydrophilic SERS substrates, making it suited for studying practical water environment sensing applications. Looking forward, modifying the AgNPs’ surface chemistry could enable the SERS sensing platforms to detect hydrophobic contaminants, such as Per- and polyfluoroalkyl substances (PFAS) or microplastics, which are emerging as societal concerning environmental pollutants.

## 4. Conclusions

In conclusion, this study successfully demonstrates a new simple fabrication method for creating thickness-tunable PDMS-based SERS substrates, achieving precise control over film thickness from sub-millimeter to 100 nm scales by utilizing D5 as an effective diluent. Further, leveraging the residual Si-H groups in PDMS, the AgNPs can be directly grown onto the PDMS surface through the reduction of $Ag^{+}$ ion in AgF solution. This enables effective SERS performance on the PDMS substrate, which is verified through R6G detection at concentrations as low as 0.1 ppm. Additionally, the hydrophobic nature of PDMS, while presenting challenges for analyte adsorption, provides a distinct advantage in mitigating surface fouling, making this substrate particularly well-suited for practical water environment sensing applications.

Following on this study, our future research will focus on (1) molding and structuring the ultra-thin PDMS film into nanophotonic architectures to enhance light–matter interactions for improved sensing performance, and (2) modifying the surface chemistry of AgNPs to target hydrophobic contaminants such as PFAS and microplastics, addressing pressing environmental challenges. Furthermore, investigating the scalability of this fabrication approach and its potential integration into wearable or biocompatible devices could be another research line to broaden its applications in health, environmental monitoring, and defense sectors.

## Data Availability

The data supporting the findings of this study are available from the corresponding authors upon reasonable request.

## Abbreviations

The following abbreviations are used in this manuscript:

SERS	Surface-Enhanced Raman Spectroscopy
PDMS	Polydimethylsiloxane
LSPR	Localized surface Plasmon Resonance
D5	Decamethylcyclopentasiloxane
EM	Electromagnetic Enhancement Mechanism
CM	Chemical Enhancement Mechanism
TEM	Transmission Electron Microscopy

## References

[1] Saletnik A., Saletnik B., Puchalski C. (2021). Overview of popular techniques of Raman spectroscopy and their potential in the study of plant tissues. Molecules.

[2] Orlando A., Franceschini F., Muscas C., Pidkova S., Bartoli M., Rovere M., Tagliaferro A. (2021). A comprehensive review on Raman spectroscopy applications. Chemosensors.

[3] Kudelski A. (2008). Analytical applications of Raman spectroscopy. Talanta.

[4] He Q., Yang W., Luo W., Wilhelm S., Weng B. (2022). Label-free differentiation of cancer and non-cancer cells based on machine-learning-algorithm-assisted fast Raman imaging. Biosensors.

[5] Fan X., Zhang H., Zhao X., Lv K., Zhu T., Xia Y., Yang C., Bai C. (2021). Three-dimensional SERS sensor based on the sandwiched G@ AgNPs@ G/PDMS film. Talanta.

[6] Ren W., Fang Y., Wang E. (2011). A binary functional substrate for enrichment and ultrasensitive SERS spectroscopic detection of folic acid using graphene oxide/Ag nanoparticle hybrids. Acs Nano.

[7] Zhang Y., Xu Z., Zhang K., Song Y., Dong B., Wang J., Yan M., Sun Q. (2024). Fabrication of Superhydrophobic–Hydrophilic Patterned Cu@ Ag Composite SERS Substrate via Femtosecond Laser. Nanomanufacturing Metrol..

[8] Nie S., Emory S.R. (1997). Probing single molecules and single nanoparticles by surface-enhanced Raman scattering. Science.

[9] Li L., Chin W.S. (2020). Rapid fabrication of a flexible and transparent Ag nanocubes@ PDMS film as a SERS substrate with high performance. ACS Appl. Mater. Interfaces.

[10] Cardinal M.F., Vander Ende E., Hackler R.A., McAnally M.O., Stair P.C., Schatz G.C., Van Duyne R.P. (2017). Expanding applications of SERS through versatile nanomaterials engineering. Chem. Soc. Rev..

[11] Li Z., Xu S.C., Zhang C., Liu X.Y., Gao S.S., Hu L.T., Guo J., Ma Y., Jiang S.Z., Si H.P. (2016). High-performance SERS substrate based on hybrid structure of graphene oxide/AgNPs/Cu film@ pyramid Si. Sci. Rep..

[12] Trantidou T., Elani Y., Parsons E., Ces O. (2017). Hydrophilic surface modification of PDMS for droplet microfluidics using a simple, quick, and robust method via PVA deposition. Microsyst. Nanoeng..

[13] Yao M., Fang J. (2012). Hydrophilic PEO-PDMS for microfluidic applications. J. Micromech. Microeng..

[14] Liu M., Sun J., Sun Y., Bock C., Chen Q. (2009). Thickness-dependent mechanical properties of polydimethylsiloxane membranes. J. Micromech. Microeng..

[15] Chen H.Y., McClelland A.A., Chen Z., Lahann J. (2008). Solventless adhesive bonding using reactive polymer coatings. Anal. Chem..

[16] Ariati R., Sales F., Souza A., Lima R.A., Ribeiro J. (2021). Polydimethylsiloxane composites characterization and its applications: A review. Polymers.

[17] González Calderón J.A., Contreras López D., Pérez E., Vallejo Montesinos J. (2020). Polysiloxanes as polymer matrices in biomedical engineering: Their interesting properties as the reason for the use in medical sciences. Polym. Bull..

[18] Guo Y., Yu J., Li C., Li Z., Pan J., Liu A., Man B., Wu T., Xiu X., Zhang C. (2018). SERS substrate based on the flexible hybrid of polydimethylsiloxane and silver colloid decorated with silver nanoparticles. Opt. Express.

[19] Repetto D., Giordano M.C., Foti A., Gucciardi P.G., Mennucci C., de Mongeot F.B. (2018). SERS amplification by ultra-dense plasmonic arrays on self-organized PDMS templates. Appl. Surf. Sci..

[20] Alyami A., Quinn A.J., Iacopino D. (2019). Flexible and transparent Surface Enhanced Raman Scattering (SERS)-Active Ag NPs/PDMS composites for in-situ detection of food contaminants. Talanta.

[21] Zhao H., Hasi W., Bao L., Liu Y., Han S., Lin D. (2018). A silver self-assembled monolayer-decorated polydimethylsiloxane flexible substrate for in situ SERS detection of low-abundance molecules. J. Raman Spectrosc..

[22] Kumar P., Khosla R., Soni M., Deva D., Sharma S.K. (2017). A highly sensitive, flexible SERS sensor for malachite green detection based on Ag decorated microstructured PDMS substrate fabricated from Taro leaf as template. Sens. Actuators B Chem..

[23] Document Viewer. https://www.dow.com/en-us/document-viewer.html?docPath=/content/dam/dcc/documents/11/11-3184-01-sylgard-184-elastomer.pdf.

[24] Lisensky G.C., Campbell D.J., Beckman K.J., Calderon C.E., Doolan P.W., Ottosen R.M., Ellis A.B. (1999). Replication and compression of surface structures with polydimethylsiloxane elastomer. J. Chem. Educ..

[25] Venkatachalam S., Hourlier D. (2019). Heat treatment of commercial Polydimethylsiloxane PDMS precursors: Part I. Towards conversion of patternable soft gels into hard ceramics. Ceram. Int..

[26] Cyclopentasiloxane (D5)—GW United Silicones. https://gwunitedsilicones.com/cyclopentasiloxane-d5/.

[27] Sun L., Kurosawa Y., Ito H., Makishima Y., Kita H., Yoshida T., Suzuri Y. (2019). Solution processing of alternating PDMS/SiOx multilayer for encapsulation of organic light emitting diodes. Org. Electron..

[28] Mariani S., La Mattina A.A., Paghi A., Strambini L., Barillaro G. (2021). Maskless Preparation of Spatially-Resolved Plasmonic Nanoparticles on Polydimethylsiloxane via In Situ Fluoride-Assisted Synthesis. Adv. Funct. Mater..

[29] Paghi A., Corsi M., Corso S., Mariani S., Barillaro G. (2022). In situ controlled and conformal coating of polydimethylsiloxane foams with silver nanoparticle networks with tunable piezo-resistive properties. Nanoscale Horizons.

[30] Swenson H., Stadie N.P. (2019). Langmuir’s theory of adsorption: A centennial review. Langmuir.

